# A just war on bugs? Ethical differences between antimalarial resistance and antibacterial resistance

**DOI:** 10.1136/bmjgh-2025-018943

**Published:** 2025-08-03

**Authors:** Tess Johnson, Lorenz von Seidlein, Phaik Yeong Cheah

**Affiliations:** 1 Pandemic Sciences Institute, University of Oxford, Oxford, UK; 2 Ethox Centre, Nuffield Department of Population Health, University of Oxford, Oxford, UK; 3 Mahidol Oxford Tropical Medicine Research Unit, Bangkok, Thailand; 4 Centre for Tropical Medicine and Global Health, University of Oxford, Oxford, UK

**Keywords:** Global Health, Environmental health, Public Health, Malaria, Interdisciplinary Research

**Summary Box**

- Antimicrobial resistance is a chronic, serious threat to humans, animals and the environment, yet measures to address it can be unjust, treating humans, animals or microbes in morally wrong ways.
- In this commentary, we argue that one factor worsening this injustice is that antibacterial resistance is the focus of many blanket interventions against antimicrobial resistance, when in fact, there are morally relevant differences between bacteria and microbes like the Plasmodium species that cause malaria in humans, which means that all microbes should not be addressed in the same way and not all interventions should receive the same level of priority.
- The differences between antibacterial resistance and antimalarial resistance imply that a just and fair way forward requires us to give antimalarial resistance a higher priority than it currently receives for research and for public health intervention.

## Introduction

 Antimicrobial resistance (AMR) refers to the ability of microbes—bacteria, viruses, fungi and parasites—to survive exposure to drugs that previously cleared them. Mostly, assessments of AMR in research papers and high-level policy documents focus on antibacterial resistance (ABR), sidelining viruses, fungi and parasites. For instance, we know that in 2019 ABR caused around 1.27 million deaths and was associated with 4.95 million deaths.^1^ We do not know how many further deaths were associated with or attributable to fungal, parasitic or viral AMR. AMR has negative implications for humans, animals and the environment, including increased morbidity and mortality and unbalanced ecosystems.^2^ AMR’s effects in the environment include exacerbating the effects of climate change, affecting animal health and reducing microbial diversity in essential reservoirs for ecosystem functioning such as soil.^3^ Approaches to addressing AMR often focus on either innovation (development of new antibiotics or alternative therapies) or stewardship (optimising use of antimicrobials). However, concerns have been raised about the injustices associated with some stewardship measures. Often, the people whose suboptimal consumption of antibiotics is targeted through stewardship measures are those who are most disadvantaged, for example, low-income parents looking for antibiotics for their children. There are clear ethical tensions between these groups’ interests and those of the general (present and future) population.^4^

In response, a ‘just transition’ approach taken from the climate change space has recently been applied to AMR^5^ to advocate for stewardship strategies that hear all voices, compensate for past harms and leave no one behind. Part of this approach involves targeting measures to the right populations and the right pathogens. There is a high burden of bacterial AMR in many low- and middle-income countries (LMICs) that experience high absolute incidence numbers of multidrug-resistant tuberculosis (MDR-TB), such as India, the Philippines and Indonesia.^6^ A just transition approach may require addressing bacterial AMR that affects the populations of LMICs as a priority. However, it may also require giving moral consideration to particular pathogenic microbes over others without merely presenting a ‘battle against the superbugs’ as a whole. Insofar as resource limitations necessitate prioritisation between interventions against AMR, it is important that we address the right resistant pathogens first. Not all microbes are equally important in the microbiome, and this may mean some should be treated in different ways. For instance, *Plasmodium* spp—the microbes causing malaria—have little or no beneficial role to play as part of balanced ecosystems, and therefore should not be considered ethically equivalent to other microbes that support balanced ecosystems or play an important role in human life and health (eg, *Escherichia coli* supports healthy gut functioning). Interventions against antimalarial resistance are therefore less ethically challenging, and perhaps as a result more promising and deserving higher priority than they currently receive in comparison to other interventions against AMR, primarily bacterial AMR. (We would also support the stronger claim that antimalarial resistance deserves higher priority than many other interventions against AMR, but acknowledging the starting point here, where antimalarial resistance is comparatively neglected in the AMR space, we find it only necessary to advocate for the weaker claim in order to support and advocate for initial changes in practice).

This is our core argument, and to make it, we begin by highlighting ethically relevant differences between particular bacterial priority pathogens and efforts to address their ABR, on the one hand, and *Plasmodium* spp and their *antimalarial* resistance, on the other. They include at-risk population and intersections with existing disadvantage; clarity of the goals of intervention and the value of more certain benefits; mortality (and therefore, potential for addressing health need); ‘one health’ issues and ease of coordination contributing to feasibility of intervention; and acceptability and effectiveness of treatment and therefore likelihood to raise ethical challenges in implementation of interventions. We use the examples of chloroquine and artemisinin resistance to illustrate our points throughout this article.

## Ethically relevant differences between bacterial pathogens’ ABR and antimalarial resistance

First, who is at risk? Malaria primarily affects sub-Saharan Africa and Southeast Asia, with African children being at highest risk of mortality.^7^ Malaria is associated with poverty. By contrast, consider those at risk from the bacterial priority pathogens whose resistance is considered high priority: the highest burden of deaths associated with bacterial AMR fell on Western sub-Saharan Africa in 2019, with around 120 deaths per 100 000 people.^1^ Deaths in Australasia were the lowest at roughly 40 per 100 000 people.^1^ Many deaths from bacterial AMR do occur in the African region. Yet, contrast this difference with the global distribution of malaria deaths: of the 597 000 deaths reported from malaria in 2023, 95% occurred in the African region.^7^ When we consider this global distribution, it is clear that a larger proportion of the population that suffers most from antimalarial resistance is based in LMICs and particularly the African region, and is likely less privileged compared with those at greatest risk from ABR. This is in part due to the geographical distribution of *Plasmodium* spp and increased mortality risk associated with antimalarial resistance compared with ABR. Box 1 elaborates on the spread of chloroquine resistance and changes to who is at risk from antimalarial resistance. Given the use of antibiotics for infection prevention during routine surgeries and the spread of bacterial disease in tertiary care settings, the harms of ABR are more broadly distributed compared with antimalarial resistance. The greater disadvantage and structural injustices already suffered by populations who are most harmed by antimalarial resistance^8^ ought to make antimalarial resistance a high priority on AMR agendas, but it is often not. This is not only unjust, but economically injudicious. The populations at risk from antimalarial resistance are younger, meaning more years of life and productivity are lost.

Box 1 **Chloroquine resistance**

Due to its ease of administration, tolerability and efficacy, chloroquine (CQ) quickly became the global first-line treatment for all malarias. The mutations required to confer CQ resistance are complex and hence infrequent. It is now thought that CQ resistance emerged de novo only four times: twice in South America (Colombia/Venezuela/Brazil) and once each in Southeast Asia (Thailand/Cambodia) and Africa (Tanzania/Kenya).^14^ Nearly 20 years passed from the first reports of CQ resistance in South America and Southeast Asia to the confirmation of CQ resistance on the African continent. The arrival of CQ resistance in Africa was initially ignored and had no immediate policy consequences.^15^ CQ remained the recommended first-line treatment throughout the turn of the century in most African countries, and many healthcare providers continued to use CQ even when faced with steadily rising failure rates.^9^ From 1990 to 1998, malaria-attributable mortality doubled in East and Southern Africa compared with the period 1982–1989, whereas non-malaria mortality was going down.^15^ Millions of lives could have been saved by a timely change in first-line treatment.

A second ethically relevant difference between antimalarial resistance and ABR is in the clarity of goals. The goal for malaria is clear: the elimination of all *Plasmodium* spp that cause human infection. This should be uncontroversial because of the clear harms and lack of any benefits from the continued existence of *Plasmodium* spp—and indeed, it was uncontroversial during early malaria eradication campaigns of the 1950s.^9^ The goals for addressing ABR are less well defined. Most bacteria should not be eliminated or eradicated. They are essential in many biological processes, including in humans, our digestion and protection against disease, and in ecosystems, for nitrogen fixation, waste breakdown and food production. Take *E. coli*: while it can become an opportunistic pathogen, it plays a role in the healthy gut to deoxygenate the gut, to produce vitamin K and to protect against pathogenic bacterial colonisation of the gut.^10^ A more nuanced approach is needed for addressing ABR, which allows for selective targeting of resistant pathogenic bacterial species, while protecting commensal and symbiotic bacteria—the goal and how to achieve it, then, are less clear than the goal of addressing antimalarial resistance and malaria through elimination.

A third key difference is in mortality and its estimation. Mortality from malaria, especially in sub-Saharan Africa, is high (with 409 000 deaths in 2019),^7^ whereas in the same region, mortality due to ABR is lower (at around a quarter of a million in 2019).^1^ It is easier to track and attribute deaths to malaria. By contrast, attributing deaths to ABR and distinguishing these from deaths merely associated with ABR is difficult—for instance, in 2019, there were 4.95 million deaths reportedly associated with ABR, but 1.27 million were strictly attributable to ABR—the process for making such determinations relied on complex modelling and comparisons of likelihood of death from drug-sensitive pathogen versus drug-resistant pathogen.^1^ Consequently, efforts to address antimalarial resistance may have more certain benefit compared with efforts to address ABR.

Fourth, many bacterial pathogens pose a ‘one health’ issue, affecting humans, animals and the environment. Take non-typhoidal *Salmonella* spp*.* Infection can affect livestock animals’ health and, through food production, humans. It can affect pets, whose treatment regimens for severe cases using ampicillin can contribute to resistance. Addressing these issues across sectors can be complex, particularly in the areas with the greatest need, where cross-sector coordination can be particularly challenging.^11^ Coordination of efforts against ABR across agricultural, health and other sectors is unfocused, with responsibilities divided between veterinary services, health ministries and industrial actors. In contrast, antimalarials are not commonly used outside their intended context. This means that targeted programmes are more common and easier to implement. There are fewer cross-sectoral or multiple-actor interventions needed against antimalarial resistance because it is not a ‘one health’ issue. This makes efforts to eliminate antimalarial resistance more feasible than efforts to address ABR.

Fifth, to minimise antimalarial resistance, strategies such as triple artemisinin combination therapies, mass drug administration and drug rotations can be implemented. While these still raise ethical issues and are controversial treatments, they do not raise the full range of questions that are present for treatments of drug-resistant bacterial infections like, say, tuberculosis.^12^ For MDR-TB, lengthy treatment courses on drugs with high toxicity are required, which are difficult for patients to adhere to. This poses ethical challenges in attributing responsibility for spreading resistant disease when avoiding doing so is highly burdensome for individuals.^6^ Similarly, ethical questions are raised by stewardship measures to address other bacterial resistance, such as restricting the use of antibiotics in farming and limiting over-the-counter access to antibiotics, all of which pose significant ethical challenges.^4^ Some of these challenges with limiting access may also apply to antimalarial stewardship, yet such issues are short lived insofar as the goal is malaria elimination. Responsibility for the problems also differs. Antimalarial resistance has emerged frequently in Southeast Asia, but >95% of the burden is concentrated in Africa. The first emergence of chloroquine resistance in *Plasmodium falciparum* in the 1960s was in the Greater Mekong subregion, and from there resistant strains spread to Africa at least six times (see box 1). This has been followed by the recent threat of spreading artemisinin resistance from Southeast Asia to Africa (see box 2). Although chloroquine use for falciparum malaria is now officially discontinued, two resistance mutations are still frequently seen in malaria-endemic countries in sub-Saharan Africa.^13^ There is a difference between those responsible and those who suffer from resistance in the case of antimalarial resistance. Multiple actors contribute to ABR globally and multiple populations are affected, resulting in a greater overlap between those morally accountable for ABR and those who suffer from it. There is, therefore, more of a restorative justice-based reason to eliminate antimalarial resistance than there is to address AMR.

Box 2 **Artemisinin resistance**

The hallmarks of the artemisinins are a very short half life measured in hours compared with days in other antimalarials, broad stage specificity, rapid reduction of parasite densities and prevention of sequestration. By combining artemisinin derivatives with longer acting antimalarials, such as mefloquine, lumefantrine or piperaquine, falciparum malaria can be cured with a 3-day regimen. Artemisinin derivatives are the cornerstone of current antimalarial treatment, both for severe malaria (injectable artesunate) and uncomplicated malaria (artemisinin combination therapies (ACTs)).

The first suggestion that ACT was no longer working as well as expected came from Cambodia in 2002. Comparing whole-genome sequences from an artemisinin-susceptible parasite line derived from Africa and clinical parasite isolates from Cambodia, which are associated with artemisinin-resistant strains, identified multiple polymorphisms associated with resistance. Many in the malaria community felt that the emergence and spread of artemisinin resistance in mainland Southeast Asia, specifically the threat of it spreading to Africa, as had occurred previously with chloroquine resistance, represented a public health emergency. In this region of low seasonal transmission and highly prevalent drug resistance, an important way to stop the spread of artemisinin resistance was to eliminate malaria.^12^ This required support for community health workers to diagnose malaria with rapid tests and treat it with ACTs and mass treatment in ‘hot spots’ as an elimination accelerator.^16^ After increased investment, falciparum malaria declined markedly in Cambodia, Lao People's Democratic Republic, Thailand and Vietnam. Still, the trend in artemisinin resistance (ART) is inexorably upward, including independent emergence in Africa.

## Conclusion

The differences between antimalarial resistance and ABR highlighted above shed light on why it is important that antimalarial resistance is given higher priority on the research and intervention agenda. Addressing antimalarial resistance should not be sidelined in broader efforts to combat AMR.

## Data Availability

There are no data in this work.
